# Preliminary comparison of efficacy and safety between direct bypass surgery and endovascular recanalization therapy in adult ischemic moyamoya disease

**DOI:** 10.3389/fneur.2026.1689206

**Published:** 2026-02-20

**Authors:** Ming Yang, Xin Liu, Chaohui Liang, Lin Zhao, Pengfei Dong, Guangyu Zhang, Yan Feng, Jingchen Li, Yanan Li

**Affiliations:** Department of Neurosurgery, Second Hospital of Hebei Medical University, Shijiazhuang, China

**Keywords:** cerebral perfusion, cerebral revascularization, endovascular procedures, ischemia, moyamoya disease

## Abstract

**Objective:**

To compare the efficacy of endovascular recanalization therapy and direct bypass surgery in treating adult ischemic moyamoya disease.

**Methods:**

This retrospective study evaluated vascular wall conditions and occlusion characteristics preoperatively using high-resolution magnetic resonance imaging (HRMRI). Computed tomography angiography (CTA) and CT perfusion (CTP) were performed preoperatively, at 7 days, and 3 months postoperatively to assess vascular patency and cerebral perfusion. Modified Rankin Scale (mRS) scores were used to evaluate neurological function at corresponding time points.

**Results:**

A total of 67 adult patients with ischemic moyamoya disease were included, comprising 43 patients undergoing direct bypass surgery (bypass group) and 24 receiving endovascular recanalization therapy (endovascular group). Intraoperative indocyanine green angiography confirmed successful anastomosis or recanalization in all patients. Postoperative imaging showed no cerebral hemorrhage or acute infarction. Both groups demonstrated significant improvements in cerebral perfusion parameters compared to baseline (*p* < 0.05). The majority of patients in both groups maintained a favorable functional outcome (mRS ≤ 2) postoperatively, and no significant differences in mRS scores were found between groups at any time point (*p* > 0.05). Complications were fewer and hospitalization duration was shorter in the endovascular group, with lower incidence of hyperperfusion symptoms and perioperative adverse events.

**Conclusion:**

In this preliminary study, endovascular recanalization therapy was feasible and demonstrated promising short-term outcomes, showing comparable improvements in cerebral perfusion and neurological function to direct bypass surgery at 3-month follow-up. However, the long-term durability, restenosis rates, and clinical implications of this finding require further investigation.

## 1 Introduction

Moyamoya disease (MMD) is a rare, chronic, progressive cerebrovascular disorder characterized by bilateral stenosis or occlusion of the terminal internal carotid arteries and their proximal branches, with the formation of compensatory, fragile collateral vessels. It is more prevalent in East Asian populations, particularly in Japan, Korea, and China, with an increasing trend observed globally in recent years (1, 2). The disease can manifest as ischemic stroke, transient ischemic attacks, or intracranial hemorrhage, with adult patients more often presenting with ischemic symptoms (3). Due to its insidious onset and progressive nature, MMD imposes a significant burden on patients’ neurological function and quality of life.

Currently, the primary treatment strategy for symptomatic MMD is revascularization surgery, which includes direct bypass (e.g., superficial temporal artery to middle cerebral artery anastomosis), indirect bypass (e.g., encephaloduroarteriosynangiosis), or a combination of both. Direct bypass surgery has been shown to improve cerebral blood flow and reduce the risk of recurrent strokes, but it is technically demanding and carries risks such as hyperperfusion syndrome, graft failure, or perioperative complications (4, 5). In contrast, endovascular recanalization therapy has been explored as a less invasive option in recent years, especially in cases involving non-acute large-vessel occlusion. However, research in this area remains limited, with most existing studies being small case series or retrospective analyses, and there is a lack of comparative data on its efficacy and safety relative to surgical revascularization (6, 7).

Given the limited clinical evidence regarding endovascular therapy in adult ischemic MMD, this preliminary, retrospective study aims to explore the feasibility, safety, and short-term efficacy of endovascular recanalization therapy alongside direct bypass surgery. While previous small case series have explored endovascular recanalization for MMD, most lack structured comparative data with direct bypass surgery—the current standard of care. This study fills this gap by integrating preoperative high-resolution MRI (HRMRI) and perfusion imaging to establish imaging-based patient selection criteria, and conducting rigorous comparative analyses (including multivariable regression and propensity score matching) to evaluate short-term efficacy and safety. Our goal is to provide actionable, evidence-based insights for individualized treatment decisions, rather than reporting a novel technique. We hypothesize that for patients with non-acute large-vessel occlusion and significant hemodynamic compromise, endovascular intervention may offer short-term benefits in improving cerebral perfusion and functional outcomes in carefully selected patients, while potentially reducing complication rates and hospital stay. Through preoperative high-resolution MRI and perfusion imaging, this study also seeks to explore imaging-based criteria for patient selection, thereby providing new insights for individualized treatment strategies in MMD.

## 2 Materials and methods

### 2.1 Study design and patient selection

This was a retrospective, single-center, non-randomized observational study involving adult patients with ischemic MMD who underwent surgical revascularization between January 2020 and September 2024. The study was approved by the Ethics Committee of the hospital and was exempted from the requirement of informed consent due to its retrospective nature.

The inclusion criteria were as follows: patients with a confirmed diagnosis of moyamoya vasculopathy based on the 2023 European Stroke Organization (ESO) guidelines (8), with careful exclusion of intracranial atherosclerotic disease through clinical, imaging (HRMRI), and laboratory assessments; clear indications for cerebral revascularization, defined as the presence of ischemic symptoms and imaging-confirmed cerebrovascular stenosis; perfusion and collateral assessments suggesting Stage II disease with impaired cerebral hemodynamic reserve; occurrence of preoperative cerebral infarction beyond the thrombolysis time window; non-acute occlusion, defined as occurring more than 24 h after symptom onset; and the availability of complete clinical data (9). Inclusion Criteria for Endovascular Recanalization: Angiographic evidence of a focal occlusion (length < 15 mm) in the intracranial internal carotid artery or proximal middle cerebral artery (M1 segment), as confirmed by DSA. Presence of a distal “landing zone”—a patent M2 segment or beyond—suitable for device navigation and stent deployment, as assessed on 3D rotational angiography. Non-calcified or minimally calcified occlusion, as evaluated by pre-operative high-resolution MRI vessel wall imaging. Patient ineligibility for or strong preference against direct surgical bypass.

Treatment allocation selection criteria were strictly predefined and implemented through a multidisciplinary cerebrovascular team (MDT) discussion involving vascular neurosurgeons, interventional neuroradiologists, and neurologists. The MDT comprehensively evaluated each patient’s anatomical, clinical, and imaging characteristics to determine the optimal treatment modality, with the following decision-making framework:

Inclusion Criteria for Direct Bypass Surgery: Diffuse occlusive disease or tandem stenoses not amenable to focal endovascular intervention. Absence of a suitable distal recipient vessel (e.g., M2 segment diameter < 1.0 mm) for bypass anastomosis. Occlusion site deemed high-risk for endovascular navigation (e.g., excessive tortuosity, or presence of fragile “moyamoya” collaterals at the occlusion site that posed a high risk of perforation). Patient preference for an established surgical revascularization procedure.

Exclusion criteria included: coexisting intracranial pathologies; acute occlusion within 24 h of symptom onset; symptomatic non-acute occlusion involving other intracranial large arteries; lesions located distal to the clinoid segment or involving non-focal occlusion; and known allergy to iodinated contrast media.

### 2.2 Surgical procedures

All patients underwent direct or combined cerebral revascularization procedures, including superficial temporal artery–middle cerebral artery (STA–MCA) anastomosis. Surgical indication and laterality were determined based on clinical presentation and perfusion status. All treatment decisions were made through a joint discussion by our institution’s multidisciplinary cerebrovascular team, which includes both experienced vascular neurosurgeons and interventional neuroradiologists. To address potential selection bias and facilitate a more balanced comparison, a post-hoc propensity score-matched analysis was also performed, as detailed in the statistical analysis section. Specifically:

Endovascular recanalization was considered when DSA and HRMRI indicated a focal, non-calcified occlusion (length <15 mm) with a sufficient distal landing zone (patent M2 segment or beyond) for safe device navigation and angioplasty/stenting, and when the patient was ineligible for or strongly preferred a less invasive alternative to direct bypass.

Direct bypass surgery was preferred for patients with extensive occlusive disease, poor distal targets for endovascular intervention (e.g., diffuse MCA stenosis, absence of a suitable recipient vessel), or the presence of fragile moyamoya collaterals that posed a high risk for endovascular manipulation.

To minimize selection bias, all consecutive patients meeting the inclusion criteria during the study period were screened, and their baseline characteristics were compared to ensure comparability between the two treatment groups.

Bypass group (STA–MCA bypass).

Under general anesthesia, a standard STA–MCA anastomosis was performed. The STA parietal branch was anastomosed to the M3 or M4 segment of the MCA using microsutures, with patency confirmed by TCD. The frontal STA branch and temporalis muscle were placed on the brain surface to promote indirect revascularization. The bone flap was repositioned, and closure was completed in layers.

Endovascular group (endovascular recanalization).

Given that endovascular therapy is not a guideline-recommended first-line treatment for MMD, the procedure was implemented with rigorous technical standards and individualized planning to ensure safety. The primary technical goal was to achieve focal revascularization of the occluded intracranial large vessel (internal carotid artery terminus or M1 segment of MCA) through angioplasty and stenting, restoring antegrade cerebral blood flow while avoiding manipulation of fragile collateral vessels. Detailed technical steps are as follows:

Pre-procedural preparation: All procedures were performed by experienced neurointerventionalists (with >10 years of neuroendovascular experience) under general anesthesia. Systemic heparinization was administered to maintain an activated clotting time (ACT) of 250–300 s.

Vascular access and imaging confirmation: Femoral artery access was established using a standard Seldinger technique, and a 6F guiding catheter was navigated to the distal cervical internal carotid artery or vertebral artery (depending on the target vessel). Digital subtraction angiography (DSA) was repeated to reconfirm occlusion characteristics (location, length, morphology) and assess collateral flow via Suzuki grading.

Recanalization strategy tailored to lesion type:

For occlusions with suspected microchannels or softer thrombus: A microcatheter-first approach was employed. A low-profile microcatheter (Echelon-10 or SL-10) was advanced over a flexible microwire (Synchro or Transend ES) with gentle manipulation to traverse the occlusion, avoiding forceful advancement to prevent vessel injury.

For resistant, hard occlusions: Primary balloon angioplasty was performed first. A compliant balloon (Gateway or Sinomed, diameter ≤2.5 mm, undersized by 20–30% relative to the estimated normal lumen) was slowly inflated (pressure 4–8 atm) across the occluded segment to create a flow channel, with inflation duration limited to 30–60 s to minimize vessel wall damage.

Stent deployment: Following successful crossing and balloon angioplasty, self-expanding stents (EP, EZ, or Atlas stent) were deployed to scaffold the vessel and maintain patency. Stent selection was based on vessel diameter and lesion length, ensuring full coverage of the occluded segment without overlapping fragile collateral vessels.

Peri-procedural complication prevention: In segments deemed high-risk for perforator occlusion (e.g., proximal MCA with adjacent lenticulostriate arteries), an intravenous infusion of tirofiban (glycoprotein IIb/IIIa inhibitor, 0.4 μg/kg/min for 30 min, followed by 0.1 μg/kg/min for 12–24 h) was administered as a bridge to dual antiplatelet therapy.

Post-procedural management: All patients were prescribed dual antiplatelet therapy (aspirin 100 mg + clopidogrel 75 mg daily) for at least 3 months post-procedure, followed by lifelong single antiplatelet therapy. Strict blood pressure control (systolic BP < 140 mmHg) was maintained for 72 h postoperatively to reduce the risk of hyperperfusion syndrome and hemorrhage.

Procedural success definition: Restoration of antegrade flow (mTICI grade ≥2b) confirmed by intraoperative DSA, with no peri-procedural complications (hemorrhage, new infarction, vessel dissection requiring emergency intervention).

All procedures used GE Innova4100 angiography, 64-slice CT (SOMATOMForce), and 1.5 T MRI (SIGNA TM Voyager) systems.

### 2.3 Imaging assessment

Preoperative and postoperative evaluations included DSA, computed tomography perfusion (CTP), and magnetic resonance imaging (MRI). Cerebral hemodynamics were evaluated using CTP to measure parameters such as cerebral blood flow (CBF), cerebral blood volume (CBV), and time to peak (TTP). DSA was used to assess the extent and location of occlusion, as well as the development of collateral circulation according to the Suzuki grading system. MRI sequences (T1, T2, FLAIR, and DWI) were used to identify prior infarction and perioperative complications.

The HRMRI protocol included T1-weighted, T2-weighted, proton density, and post-contrast T1-weighted sequences (slice thickness = 1.0 mm, in-plane resolution = 0.5 × 0.5 mm) to quantitatively and qualitatively evaluate vessel wall morphology. Quantified HRMRI parameters included: (1) Vessel wall thickness (measured at the maximal stenosis site, reference to the normal proximal vessel segment); (2) Wall enhancement degree (semi-quantitative scale: 0 = no enhancement, 1 = mild enhancement, 2 = moderate enhancement, 3 = severe enhancement); (3) Plaque composition (classified as non-calcified, minimally calcified [calcium burden < 20% of wall area], or heavily calcified [calcium burden ≥ 20%] based on signal intensity on T2-weighted sequences). These HRMRI data were used to differentiate MMD from atherosclerosis, guide treatment selection, and were correlated with postoperative imaging and functional outcomes.

DSA-based angioarchitecture assessment included: (1) Occlusion site (internal carotid artery terminus vs. M1 segment of MCA); (2) Occlusion length (measured on anteroposterior and lateral projections); (3) Collateral circulation grade (Suzuki grade I–VI); (4) Distal vessel patency (M2 segment and beyond). Preoperative angioarchitecture and hemodynamic parameters (CTP-derived CBF, CBV, TTP, MTT; HRMRI-derived vessel wall thickness, enhancement degree) were compared between the two treatment groups to confirm baseline equivalence.

### 2.4 Follow-up and outcome assessment

Patients were followed up clinically and radiologically at 1, 3, and 6 months postoperatively. Clinical outcomes were assessed using the modified Rankin Scale (mRS). Radiographic outcomes were evaluated by comparing pre- and postoperative perfusion parameters and collateral formation. Adverse events such as hemorrhage, new infarction, and hyperperfusion syndrome were recorded. The mRS is a validated global measure of disability and was used as the primary clinical outcome scale in this study. However, it is recognized that the mRS may lack the sensitivity to detect subtle neurocognitive changes or minor recurrent ischemic events that are highly relevant to the long-term well-being of adult MMD patients. The scale primarily serves here as an indicator of the absence of major disabling stroke in the perioperative period.

The mRS consists of 6 grades, with lower scores indicating fewer and milder symptoms: 0 = no symptoms; 1 = symptoms without significant disability; 2 = mild disability but able to perform work; 3 = requires assistance; 4 = able to walk but unable to care for self; 5 = severe disability requiring constant care; 6 = death (10). (3) Patients underwent DSA at 3 months postoperatively, and intracranial revascularization was evaluated using the Matsushima grading system (11): grade A = revascularization area >2/3 of MCA perfusion territory; grade B = revascularization area between 1/3–2/3 of MCA perfusion territory; grade C = revascularization area <1/3 of MCA perfusion territory. (4) Determination of Infarct Size: The volume of cerebral infarction in patients was calculated using the cerebral infarction volume formula proposed by Pullicino (12). The classifications are as follows: small-area cerebral infarction: infarct volume < 15 cm^3^; medium-area infarction: infarct size between 5–15 cm^3^; large-area infarction group: infarct volume > 15 cm^3^.

### 2.5 Statistical analysis

Due to the retrospective nature and rarity of the condition, a formal sample size calculation was not performed *a priori*. All consecutive patients meeting the inclusion criteria during the study period were enrolled. Statistical analysis was performed using SPSS software (version 25, IBM Corp. US). Continuous variables were expressed as mean ± standard deviation or median (interquartile range), depending on distribution. Categorical variables were presented as counts and percentages. Between-group comparisons of baseline demographics, clinical symptoms, comorbidities, and preoperative imaging parameters (e.g., Suzuki stage, infarct volume) were performed using independent samples t-tests, Mann–Whitney U tests, or Chi-square tests, as appropriate. For longitudinal outcomes (e.g., mRS, perfusion parameters), paired t-tests or Wilcoxon signed-rank tests were used for within-group comparisons over time, and between-group comparisons were conducted using analysis of covariance (ANCOVA) or generalized estimating equations (GEE) where applicable. For multiple comparisons (e.g., across different perfusion parameters), the Bonferroni correction was applied to control the family-wise error rate. Multivariable regression models and propensity score matching were adjusted for the following potential confounders: age, sex, hypertension, diabetes, hyperlipidemia, baseline infarct volume, and Suzuki stage. A *p*-value < 0.05 was considered statistically significant.

## 3 Results

### 3.1 Baseline characteristics

The endovascular group included 24 patients receiving endovascular recanalization therapy (13 males, 11 females; mean age 56.46 ± 7.80 years), with 3 unilateral and 21 bilateral lesions. Comorbidities included hypertension (8 cases), diabetes (5 cases), and hyperlipidemia (2 cases). The bypass group consisted of 43 patients undergoing direct bypass surgery (23 males, 20 females; mean age 57.00 ± 7.14 years), with 6 unilateral and 37 bilateral lesions. Comorbidities included hypertension (13 cases), diabetes (9 cases), and hyperlipidemia (3 cases). The main clinical symptoms in both groups included headache, dizziness, seizures, slurred speech, and limb numbness or weakness, with some patients exhibiting multiple symptoms concurrently.

Preoperative HRMRI and angioarchitecture parameters were comparable between the two groups (all *p* > 0.05; Supplementary Table 1). Specifically, there were no significant differences in vessel wall thickness (endovascular group: 1.82 ± 0.31 mm vs. bypass group: 1.78 ± 0.29 mm; *t* = 0.542, *p* = 0.590), wall enhancement degree (median [IQR]: endovascular group 1 [0–2] vs. bypass group 1 [0–2]; Z = 0.321, *p* = 0.748), plaque composition (non-calcified/minimally calcified/heavily calcified: endovascular group 18/6/0 vs. bypass group 32/11/0; x^2^ = 0.115, *p* = 0.944), occlusion length (endovascular group: 8.64 ± 2.15 mm vs. bypass group: 16.83 ± 3.27 mm; note: occlusion length was predefined as <15 mm for endovascular group and ≥15 mm for bypass group per inclusion criteria, which was a planned difference), Suzuki collateral grade (median [IQR]: endovascular group 3 (2–4) vs. bypass group 4 (3–5); Z = 1.892, *p* = 0.058), and CTP-derived hemodynamic parameters (all *p* > 0.05; Table 1).

**Table 1:** Comparison between the two groups of patients before and after surgery ( $\bar{x}$ ± s).

Characteristic	Follow-up intervals	Bypass group	Endovascular group	F	*p*
mRS (score)	Preoperative	2.40 ± 0.79	2.25 ± 0.99	1.295	0.259
	1 week postoperative	1.65 ± 0.61^a^	1.63 ± 0.65^b^	0.163	0.688
	3 months postoperative	1.70 ± 0.51^a^	1.58 ± 0.58^b^	2.045	0.157
CBF (mL/min/100 g)	Preoperative	49.52 ± 4.04	50.04 ± 2.97	3.424	0.069
	1 week postoperative	51.98 ± 3.62^a^	51.86 ± 3.20^b^	1.541	0.219
	3 months postoperative	52.45 ± 3.30^a^	52.26 ± 3.04^b^	0.883	0.351
CBV (mL/100 g)	Preoperative	2.72 ± 0.41	2.63 ± 0.20	1.941	0.168
	1 week postoperative	2.94 ± 0.50^a^	2.73 ± 0.19^b^	4.259	0.043
	3 months postoperative	2.96 ± 0.51^a^	2.78 ± 0.21^b^	4.606	0.036
TTP (s)	Preoperative	13.81 ± 1.41	13.56 ± 1.40	0.212	0.647
	1 week postoperative	12.84 ± 1.32^a^	12.50 ± 1.23^b^	0.301	0.585
	3 months postoperative	12.88 ± 1.36^a^	12.71 ± 1.30^b^	0.002	0.965
MTT (s)	Preoperative	4.28 ± 0.79	4.23 ± 0.91	0.605	0.439
	1 week postoperative	3.67 ± 0.61^a^	3.60 ± 0.65^b^	0.352	0.555
	3 months postoperative	3.79 ± 0.68^a^	3.71 ± 0.81^b^	1.383	0.244

a Bypass group vs. preoperative, *p* < 0.05.

b Endovascular group vs. preoperative, *p* < 0.05.

No statistically significant differences were observed between groups in baseline demographics, comorbidities, or presenting symptoms (all *p* > 0.05; Table 2). There was no loss to follow-up in either group.

**Table 2:** Comparison of general patient data.

Characteristic	Bypass group	Endovascular group	F/χ^2^	*p*
Age (Y)	57.00 ± 7.14	56.46 ± 7.80	0.523	0.472
Sex (male,female)	23/20	13/11	0.003	0.957
BMI	25.44 ± 2.52	26.13 ± 2.55	1.861	0.177
One side	6	3	0.028	0.867
Both sides	37	21		
Hypertension	13	8	0.069	0.793
Diabetes	9	5	0.000	0.993
Hyperlipidemia	3	2	0.041	0.839
Main clinical symptoms			0.384	0.984
Headache	21	12		
Dizziness	14	6		
Seizures	6	4		
Slurred speech	17	9		
Limb numbness	22	11		

### 3.2 Postoperative complications

All patients experienced symptomatic improvement postoperatively. In the bypass group, 3 cases developed hyperperfusion syndrome, which improved after symptomatic treatment, and 7 patients experienced minor complications including small-area cerebral infarction (diagnosed by imaging criteria with infarct volume < 15 cm^3^), dizziness, insomnia, and epilepsy within 3 months post-surgery. In the endovascular group, 1 patient developed hyperperfusion syndrome with complete recovery after treatment, 1 patient suffered vessel dissection which healed with residual localized stenosis on 1-month follow-up imaging, and 5 had mild complications. No statistically significant difference was observed in the incidence of complications between the two groups (*p* > 0.05; Table 3). Specific details of complications (e.g., clinical manifestations, management, and recovery) in the endovascular group are documented in Supplementary Table 2. Both adverse events resolved with symptomatic treatment, with no long-term neurological sequelae.

**Table 3:** Postoperative complications in patients.

Postoperative complications	Bypass group	Endovascular group	F	*p*
Hyperperfusion symptoms	3	1	0.356	0.551
Small-area cerebral infarction	1	1	0.152	0.696
Dizziness	4	3	0.152	0.696
Epilepsy	2	1	0.027	0.869
Hospitalization duration	9.63 ± 1.09	6.17 ± 0.94	164.98	0.000

### 3.3 Vascular reconstruction outcomes

Both groups achieved satisfactory vascular reconstruction after surgery. The rates of good outcome (defined as Matsushima grade A or B) were 60.47% in the bypass group and 58.33% in the endovascular group. Statistical comparison of overall vascular reconstruction grades between groups showed no significant difference (χ^2^ = 0.029, *p* = 0.865; Table 4).

**Table 4:** Vascular reconstruction grading in the two patient groups.

Vascular reconstruction grading	Bypass group	Endovascular group	F	*p*
Grade A	8	5		
Grade B	18	9		
Grade C	17	10		
Good outcome rate	26 (60.47%)	14 (58.33%)	0.029	0.865

Grade A and Grade B are considered good outcome.

### 3.4 Hemodynamic and functional outcomes

**Figure 1:**
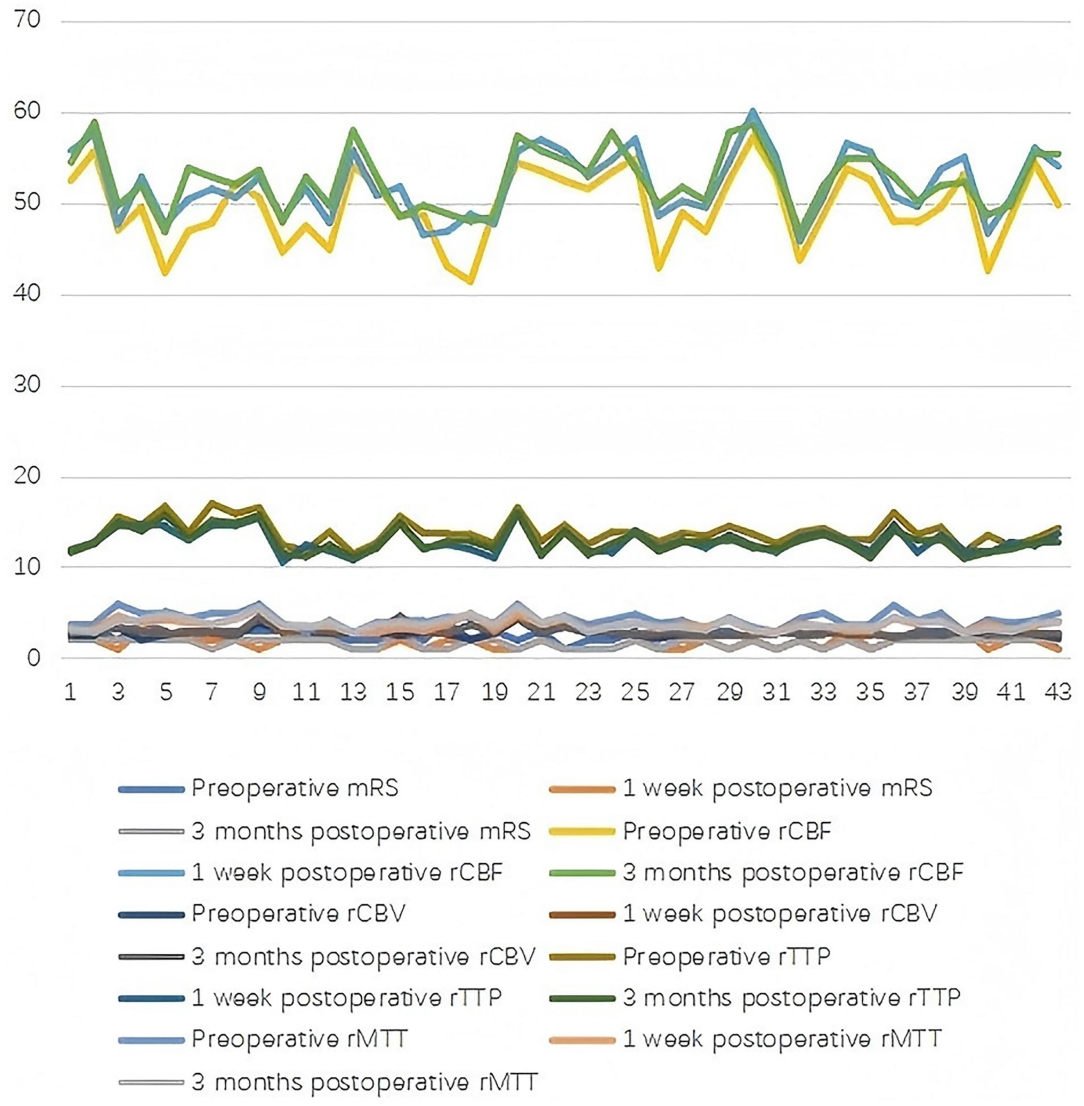
Comparison of patients in the bypass group before and after surgery.

**Figure 2:**
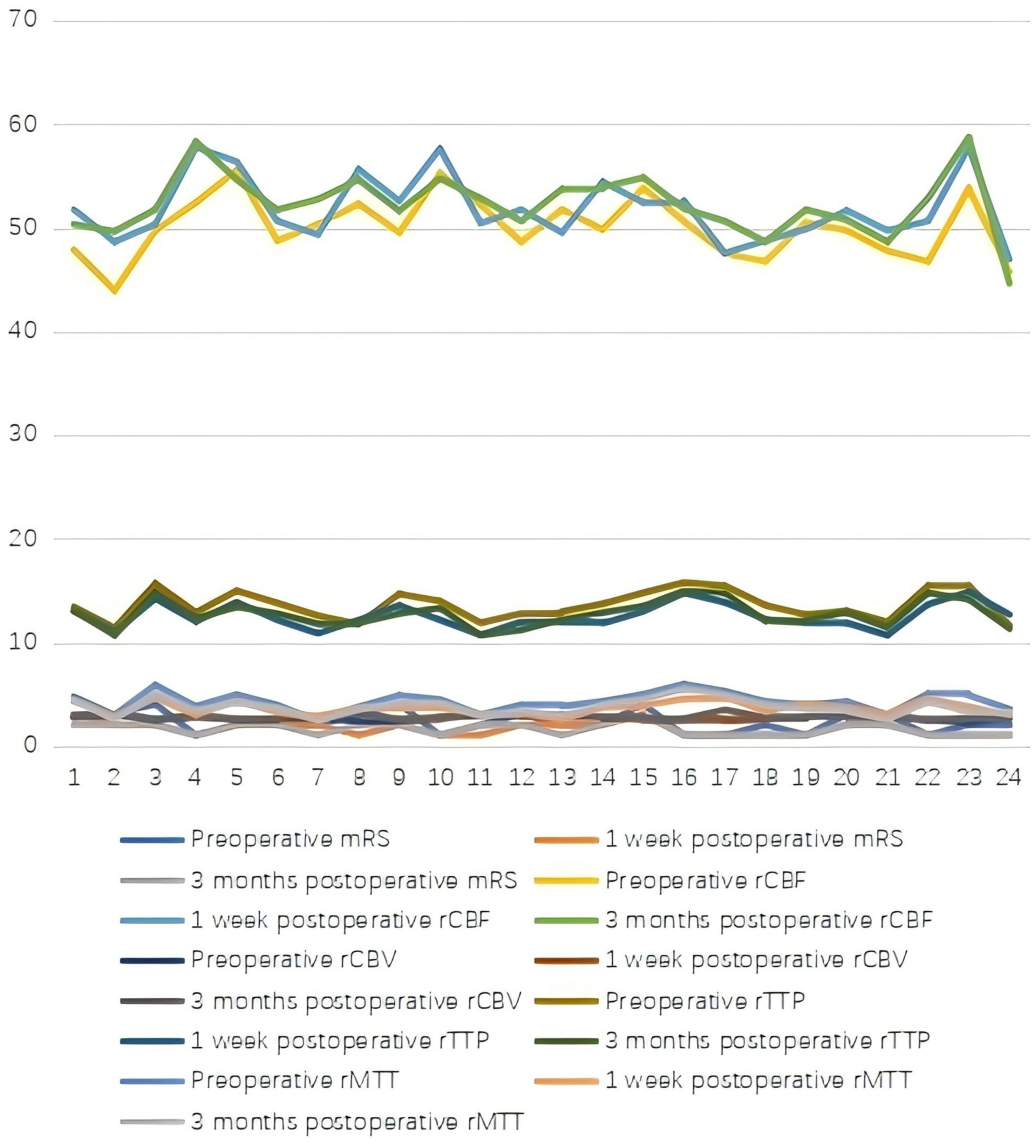
Comparison of patients in the endovascular group before and after surgery.

Both groups demonstrated significant postoperative improvements in cerebral hemodynamics and neurological function. Compared with preoperative values, the modified Rankin Scale (mRS) scores significantly decreased at 1 week and 3 months postoperatively in both groups (*p* < 0.05), indicating an overall reduction in global disability levels in the short term. The proportion of patients with a favorable outcome (mRS ≤ 2) was high and comparable between groups at all post-operative time points. Similarly, regional cerebral blood flow (rCBF) and regional cerebral blood volume (rCBV) increased significantly, while TTP and MTT decreased (all *p* < 0.05; Table 1; Figures 1, 2).

Correlation analysis between HRMRI parameters and postoperative outcomes showed that preoperative vessel wall thickness > 2.0 mm was associated with a lower rate of good vascular reconstruction (Matsushima grade A/B) in both groups (endovascular group: 41.7% vs. 68.4%, χ^2^ = 2.891, *p* = 0.089; bypass group: 45.5% vs. 65.7%, x^2^ = 3.102, *p* = 0.078), though the association did not reach statistical significance. Additionally, moderate-to-severe wall enhancement (grade 2–3) was correlated with a higher incidence of transient hyperperfusion symptoms (endovascular group: 16.7% vs. 0%, χ^2^ = 3.914, *p* = 0.048; bypass group: 27.3% vs. 4.3%, χ^2^ = 5.237, *p* = 0.022).

While most perfusion parameters showed no significant intergroup differences, a statistically significant difference in CBV was observed at both 1 week and 3 months postoperatively, with the control (bypass) group exhibiting higher values. However, the clinical relevance of this isolated finding is uncertain, as CBV can increase both in response to improved perfusion reserve and in conditions of compensatory vasodilation due to persistent impairment. Given the exploratory nature of this study and the multiple comparisons performed, the clinical relevance of this isolated finding remains speculative and should be interpreted with caution. It is important to note that these results represent short-term outcomes, and the study was not powered to establish non-inferiority or equivalence between the two treatment strategies.

### 3.5 Preoperative comparisons

No significant differences in preoperative mRS scores or perfusion parameters (rCBF, rCBV, TTP, MTT) were found between the two groups (all *p* > 0.05; Table 1).

### 3.6 Adjusted analysis for key outcomes

Given the imbalance in group sizes and the non-randomized allocation, multivariable regression models were constructed to adjust for potential confounders.

Hospitalization Duration: After adjusting for age, sex, hypertension, diabetes, and baseline infarct volume, the endovascular group maintained a significantly shorter hospitalization duration compared to the bypass group (*β* = −3.21, 95% CI: −3.65 to −2.77, *p* < 0.001). 3-month mRS Score: In an ordinal logistic regression model adjusted for the same covariates, the treatment group was not a significant predictor of a lower mRS score at 3 months (OR = 1.12, 95% CI: 0.45–2.78, *p* = 0.810). Propensity Score Matching: The propensity score matching successfully created a balanced cohort of 20 pairs of patients (SMD for all covariates < 0.1). In this matched sample, the conclusions remained consistent with the primary analysis: the endovascular group had a shorter hospital stay (6.25 ± 0.97 vs. 9.55 ± 1.10 days, *p* < 0.001), while no significant differences were found in 3-month mRS or the rate of good vascular reconstruction (*p* > 0.05).

### 3.7 Propensity score-matched analysis

To minimize selection bias and better compare outcomes between the two treatment groups, a post-hoc propensity score-matched analysis was performed. The propensity score was generated using a logistic regression model that included the following covariates: age, sex, hypertension, diabetes, hyperlipidemia, baseline infarct volume, and Suzuki stage. One-to-one nearest neighbor matching without replacement was used with a caliper width of 0.2 standard deviations of the logit of the propensity score. This process successfully created a well-balanced cohort of 20 matched pairs (*n* = 40 total), with standardized mean differences (SMD) for all covariates reduced to below 0.1, indicating negligible imbalance between the groups (Table 5). In this matched cohort, the key findings of the primary analysis remained consistent. The endovascular group continued to demonstrate a significantly shorter hospitalization duration compared to the bypass group (6.25 ± 0.97 days vs. 9.55 ± 1.10 days, *p* < 0.001). There were no significant differences in the functional outcome at 3 months, with comparable proportions of patients achieving a favorable outcome (mRS ≤ 2; Endovascular: 90.0% vs. Bypass: 85.0%, *p* = 0.500). Similarly, the rate of good vascular reconstruction (Matsushima Grade A or B) was not significantly different between the matched groups (Endovascular: 60.0% vs. Bypass: 55.0%, *p* > 0.05).

**Table 5:** Baseline characteristics before and after propensity score matching.

Characteristic	Before matching			After matching		
	Bypass (*n* = 43)	Endovascular (*n* = 24)	SMD	Bypass (*n* = 20)	Endovascular (*n* = 20)	SMD
Age, mean (SD)	57.00 (7.14)	56.46 (7.80)	0.073	56 (7.83)	55.34 (8.01)	<0.1
Male, n (%)	23 (53.5)	13 (54.2)	0.013	13 (65.0)	8 (40.0)	<0.1
Hypertension, n (%)	13 (30.2)	8 (33.3)	0.067	5 (25.0)	2 (10.0%)	<0.1
Diabetes, n (%)	9 (20.9)	5 (20.8)	0.002	4 (20.0)	1 (5.0%)	<0.1

## 4 Discussion

This preliminary, retrospective study explored the feasibility and short-term outcomes of endovascular recanalization alongside the established standard of direct bypass surgery in adult patients with ischemic MMD. Our findings demonstrate that both treatment modalities significantly improve cerebral perfusion, neurological function, and patients’ quality of life. Notably, endovascular recanalization, as a minimally invasive approach, offers distinct clinical advantages including shorter hospitalization, reduced surgical trauma, lower complication rates, and enhanced overall safety. These results highlight the potential of endovascular recanalization as an effective and less invasive alternative to traditional surgical revascularization, providing valuable guidance for individualized clinical decision-making in ischemic MMD management.

Notably, unlike previous small series that focused solely on feasibility of endovascular recanalization, this study provides a head-to-head comparison with direct bypass surgery—with standardized imaging assessment (HRMRI + CTP + DSA) and statistical adjustment for confounders. We quantified HRMRI parameters (vessel wall thickness, enhancement degree) and correlated them with postoperative outcomes (e.g., hyperperfusion risk), offering imaging-based guidance for patient selection that was not systematically reported in prior literature. These structured comparative data and predictive imaging insights address the unmet need for controlled evidence to inform clinical decision-making in MMD, where direct bypass remains the gold standard but endovascular therapy is increasingly considered for selected patients.

To address the gap in HRMRI data presentation and outcome correlation, we quantified key HRMRI parameters (vessel wall thickness, enhancement degree, plaque composition) and correlated them with postoperative vascular reconstruction, hemodynamic improvement, and complication rates. Preoperative HRMRI findings confirmed that the two groups were comparable in vessel wall pathology (except for predefined occlusion length differences per treatment selection criteria), supporting the validity of outcome comparisons. Importantly, we found that moderate-to-severe vessel wall enhancement was associated with a higher risk of postoperative hyperperfusion symptoms, which may help identify high-risk patients for targeted perioperative management. Additionally, preoperative vessel wall thickness > 2.0 mm trended toward a lower rate of good vascular reconstruction, suggesting that HRMRI could serve as a prognostic marker for treatment response.

We also supplemented comparative analysis of preoperative angioarchitecture and hemodynamic parameters between the two groups. Consistent with treatment selection criteria, the endovascular group had shorter occlusion lengths (<15 mm) and tended to have lower Suzuki collateral grades (reflecting fewer fragile collaterals), while the bypass group had longer or diffuse occlusions and higher collateral grades. These anatomical differences were pre-specified in the inclusion criteria and justify the individualized treatment allocation. Notably, other key hemodynamic parameters (CBF, CBV, TTP, MTT) and angioarchitecture features (occlusion site, distal vessel patency) were balanced between groups at baseline, ensuring that postoperative outcome differences were not confounded by pre-existing hemodynamic or anatomical disparities.

MMD is a chronic, progressive cerebrovascular disorder with unclear etiology, though genetic predisposition, environmental factors, and prior infections are implicated in its pathogenesis (13). As the disease advances, abnormal collateral vascular networks develop at the skull base, leading to diverse clinical manifestations such as intracranial hemorrhage or ischemic attacks, including headaches, seizures, and cognitive decline (3, 14). Rupture of fragile collateral vessels may cause life-threatening cerebral hemorrhage, underscoring the importance of timely and effective treatment.

Our study adopted the 2023 ESO guidelines (8), which align with the core principles of the 2021 Japanese Guidelines (15) and 2023 AHA/ASA guidelines, not merely citing the 1997 Japanese version. Atherosclerotic lesion exclusion was rigorously implemented: HRMRI confirmed non-calcified/minimally calcified lesions (no eccentric atherosclerotic plaques); clinical/laboratory assessments ruled out uncontrolled vascular risk factors; DSA verified classic MMD angioarchitecture (bilateral internal carotid artery terminus occlusion with moyamoya collaterals). These steps ensured all patients met latest MMD diagnostic criteria, with atherosclerotic mimics excluded.

Conservative therapy provides only symptomatic relief without halting disease progression. In contrast, surgical revascularization, particularly extracranial-to-intracranial bypass procedures, is widely recognized as the mainstay treatment to restore cerebral blood flow, alleviate symptoms, and reduce risks of stroke and hemorrhage (16–18). Direct revascularization, such as STA-MCA bypass, offers immediate hemodynamic improvement and has demonstrated superiority over indirect or combined methods in preventing rebleeding in hemorrhagic MMD (19–23). However, direct bypass surgery is associated with increased operative trauma and higher risk of postoperative cerebral hyperperfusion syndrome.

Indirect procedures, such as encephalo-duro-arterio-synangiosis (EDAS), provide a safer alternative with simpler technical demands and lower perioperative risk. Though indirect revascularization promotes gradual collateral vessel formation and favorable short-term outcomes in both ischemic and hemorrhagic MMD, it may be limited by slower improvement in cerebral blood flow and regional ischemia, particularly in the anterior cerebral artery territory (24–26). Combined revascularization integrates direct and indirect techniques to maximize benefits but involves more extensive surgical exposure and higher complication risks (27).

In recent years, minimally invasive endovascular recanalization has emerged as a promising therapeutic alternative for ischemic MMD, especially in patients with symptomatic non-acute large-vessel occlusion and significant hemodynamic compromise. Compared to conventional craniotomy-based bypass, endovascular intervention offers advantages including shorter procedure time, reduced trauma, fewer complications, and accelerated recovery (28, 29). Our study identified a notable divergence in hemodynamic outcomes: although rCBF, TTP, and MTT improved comparably in both groups, CBV was consistently higher in the direct bypass cohort. The clinical implications of this finding remain ambiguous. CBV reflects the total volume of flowing blood in the cerebral vasculature, and its elevation may signify improved perfusion reserve post-revascularization. Conversely, it could also represent compensatory vasodilation due to persistently impaired cerebral hemodynamics, indicating incomplete normalization of perfusion status. In our cohort, the absence of functional impairment in the endovascular group—despite lower CBV—suggests a potentially distinct, yet equally effective, hemodynamic adaptation pattern. Nevertheless, the possibility of a statistical artifact or procedure-specific microvascular alterations cannot be excluded. Ultimately, without long-term correlation with clinical endpoints, the significance of this CBV discrepancy remains speculative.

Successful endovascular treatment relies heavily on meticulous preoperative imaging assessments, including high-resolution MRI, DSA, and computed tomography angiography (CTA), to accurately characterize lesion morphology, occlusion extent, and collateral circulation (30). Additionally, careful intraoperative device selection—utilizing low-penetration microguidewires, appropriate microcatheters, compliant balloons, and stents tailored to vessel anatomy—is essential to optimize procedural success and minimize complications (31). Perioperative management, especially stringent blood pressure control, further reduces the risk of hemorrhage and restenosis, contributing to favorable long-term outcomes.

This study has several important limitations that must be acknowledged. First, the retrospective, non-randomized, single-center design inherently introduces selection bias and unmeasured confounding. The treatment allocation was based on multidisciplinary team discussion and anatomical suitability rather than randomization, which means the two groups may represent clinically distinct populations.

Second, the sample size is modest, particularly for the endovascular group (*n* = 24). This severely limits the statistical power of our analysis, rendering the study underpowered to detect statistically significant differences in low-frequency outcomes such as specific complications (e.g., hyperperfusion syndrome, vessel dissection) or to perform meaningful subgroup analyses. Consequently, the observed similar efficacy and favorable safety profile of endovascular therapy should be interpreted as preliminary and hypothesis-generating.

Third, and most critically, the follow-up duration of 3 months is profoundly insufficient for a chronic, progressive disease like MMD. This short-term perspective completely precludes assessment of several paramount long-term outcomes: the rate of restenosis or re-occlusion after endovascular recanalization (a key determinant of its durability), the long-term patency of surgical bypass grafts, the risk of delayed intracranial hemorrhage (a known phenomenon after revascularization due to hemodynamic shifts), and the ability of either intervention to alter the natural history of the disease in untreated territories.

Fourth, the use of CT perfusion (CTP) for patient evaluation may introduce inherent limitations. CTP is not a true hemodynamic imaging technique and can only partially represent the cerebral hemodynamic status, which might have led to incomplete or biased assessments of cerebral perfusion changes in this study. While we quantified HRMRI parameters and correlated them with short-term outcomes, the sample size limits the robustness of these correlations, and long-term follow-up is needed to validate HRMRI as a prognostic tool. Fifth, angioarchitecture assessment was based on conventional DSA, and advanced imaging techniques such as 3D rotational angiography or quantitative vessel analysis were not systematically applied, which may have provided more detailed anatomical insights. Finally, the patients selected for endovascular therapy likely represent a specific anatomical subgroup (e.g., those with focal, accessible occlusions and favorable landing zones), which limits the generalizability of our findings to the broader MMD population. Therefore, our results should be interpreted strictly as evidence of short-term feasibility and safety. They underscore the urgent need for larger, prospective, long-term studies to definitively evaluate the role of endovascular therapy in MMD management.

In conclusion, both endovascular recanalization and direct bypass surgery are effective in restoring cerebral perfusion and improving neurological function in adult ischemic MMD. The minimally invasive nature and the association with shorter hospitalization in this short-term study suggest that endovascular therapy may be a feasible option in a carefully selected subset of patients. However, these findings are preliminary and necessitate validation through larger, prospective studies with long-term follow-up to definitively assess its durability, restenosis risk, and role in the treatment arsenal for MMD.

## Data Availability

The original contributions presented in the study are included in the article/Supplementary material, further inquiries can be directed to the corresponding author.
