# Supplementary material for: Preliminary comparison of efficacy and safety between direct bypass surgery and endovascular recanalization therapy in adult ischemic moyamoya disease
Source: Front Neurol. 2026 Feb 20;17:1689206. doi: 10.3389/fneur.2026.1689206 (PMC12962938; doi:10.3389/fneur.2026.1689206)
Supplement: Supplementary file 1 [file Table_1.DOCX]

Supplementary Table 1: Comparison of Preoperative HRMRI and Angioarchitecture Parameters Between Two Groups

| **Parameter** | **Endovascular Group (n=24)** | **Bypass Group (n=43)** | **Statistic** | **P** |
| --- | --- | --- | --- | --- |
| HRMRI Parameters |  |  |  |  |
| Vessel wall thickness (mm) | 1.82 ± 0.31 | 1.78 ± 0.29 | t = 0.542 | 0.590 |
| Wall enhancement degree (median [IQR]) | 1 [0–2] | 1 [0–2] | Z = 0.321 | 0.748 |
| Plaque composition (n, %) |  |  | χ² = 0.115 | 0.944 |
| Non-calcified | 18 (75.0%) | 32 (74.4%) |  |  |
| Minimally calcified | 6 (25.0%) | 11 (25.6%) |  |  |
| Heavily calcified | 0 (0%) | 0 (0%) |  |  |
| Angioarchitecture Parameters |  |  |  |  |
| Occlusion site (n, %) |  | χ² = 1.028 | 0.311 |  |
| ICA terminus | 15 (62.5%) | 29 (67.4%) |  |  |
| M1 segment | 9 (37.5%) | 14 (32.6%) |  |  |
| Occlusion length (mm) | 8.64 ± 2.15 | 16.83 ± 3.27 | t = 12.893 | <0.001* |
| Suzuki collateral grade (median [IQR]) | 3 [2–4] | 4 [3–5] | Z = 1.892 | 0.058 |
| Distal vessel patency (M2+, n, %) | 24 (100%) | 41 (95.3%) | χ² = 1.987 | 0.159 |

*Note: Statistically significant difference due to predefined inclusion criteria (occlusion length <15 mm for endovascular group, ≥15 mm for bypass group). IQR = interquartile range; ICA = internal carotid artery; MCA = middle cerebral artery.
