# Supplementary material for: Preliminary comparison of efficacy and safety between direct bypass surgery and endovascular recanalization therapy in adult ischemic moyamoya disease
Source: Front Neurol. 2026 Feb 20;17:1689206. doi: 10.3389/fneur.2026.1689206 (PMC12962938; doi:10.3389/fneur.2026.1689206)
Supplement: Supplementary file 2 [file Table_2.DOCX]

Supplementary Table 2 Individual Details of 24 Patients Undergoing Endovascular Recanalization Therapy

| **Patient ID** | **Age (Y)** | **Sex** | **Comorbidities** | **Preoperative DSA Findings** |  |  |  | **Intraoperative Details** |  |  | **Postoperative Outcomes** |  |  |
| --- | --- | --- | --- | --- | --- | --- | --- | --- | --- | --- | --- | --- | --- |
|  |  |  |  | Occlusion Location | Occlusion Length (mm) | Suzuki Collateral Grade | Distal Landing Zone (M2 Diameter, mm) | Recanalization Strategy | Device Selection | Post-procedure mTICI Grade | 3-Month Vascular Patency (DSA) | Complications | 3-Month mRS Score |
| 1 | 52 | M | None | ICA terminus | 7.2 | 3 | 1.4 | Microcatheter-first | Echelon-10 + Synchro + EP stent | 2b | Patent | None | 1 |
| 2 | 61 | F | Hypertension | M1 segment | 9.5 | 2 | 1.2 | Microcatheter-first | SL-10 + Transend ES + EZ stent | 2b | Patent | None | 1 |
| 3 | 55 | M | Diabetes | ICA terminus | 12.1 | 4 | 1.5 | Balloon angioplasty + stenting | Gateway (2.0mm) + Atlas stent | 2b | Patent | None | 2 |
| 4 | 49 | F | None | M1 segment | 6.8 | 3 | 1.3 | Microcatheter-first | Echelon-10 + Synchro + EP stent | 2b | Patent | None | 1 |
| 5 | 58 | M | Hypertension + Hyperlipidemia | ICA terminus | 14.3 | 4 | 1.6 | Balloon angioplasty + stenting | Sinomed (2.5mm) + EZ stent | 2b | Patent | None | 2 |
| 6 | 63 | F | Diabetes | M1 segment | 8.9 | 3 | 1.1 | Microcatheter-first | SL-10 + Transend ES + EP stent | 2b | Patent | None | 1 |
| 7 | 50 | M | None | ICA terminus | 10.4 | 2 | 1.4 | Balloon angioplasty + stenting | Gateway (2.0mm) + Atlas stent | 2b | Patent | None | 1 |
| 8 | 57 | F | Hypertension | M1 segment | 7.5 | 3 | 1.2 | Microcatheter-first | Echelon-10 + Synchro + EZ stent | 2b | Patent | None | 2 |
| 9 | 60 | M | None | ICA terminus | 13.2 | 4 | 1.5 | Balloon angioplasty + stenting | Sinomed (2.5mm) + EP stent | 2b | Patent | None | 1 |
| 10 | 53 | F | None | M1 segment | 9.1 | 2 | 1.3 | Microcatheter-first | SL-10 + Transend ES + Atlas stent | 2b | Patent | None | 1 |
| 11 | 59 | M | Diabetes | ICA terminus | 8.3 | 3 | 1.4 | Microcatheter-first | Echelon-10 + Synchro + EZ stent | 2b | Patent | None | 2 |
| 12 | 47 | F | None | M1 segment | 6.5 | 3 | 1.1 | Microcatheter-first | SL-10 + Transend ES + EP stent | 2b | Patent | None | 1 |
| 13 | 56 | M | Hypertension | ICA terminus | 11.7 | 4 | 1.6 | Balloon angioplasty + stenting | Gateway (2.0mm) + EZ stent | 2b | Patent | None | 2 |
| 14 | 62 | F | None | M1 segment | 10.8 | 3 | 1.3 | Balloon angioplasty + stenting | Sinomed (2.5mm) + Atlas stent | 2b | Patent | None | 1 |
| 15 | 54 | M | None | ICA terminus | 7.9 | 2 | 1.4 | Microcatheter-first | Echelon-10 + Synchro + EP stent | 2b | Patent | None | 1 |
| 16 | 51 | F | Diabetes | M1 segment | 8.6 | 3 | 1.2 | Microcatheter-first | SL-10 + Transend ES + EZ stent | 2b | Patent | None | 2 |
| 17 | 64 | M | Hypertension | ICA terminus | 14.8 | 4 | 1.7 | Balloon angioplasty + stenting | Gateway (2.0mm) + Atlas stent | 2b | Patent | None | 2 |
| 18 | 48 | F | None | M1 segment | 7.3 | 2 | 1.3 | Microcatheter-first | Echelon-10 + Synchro + EP stent | 2b | Patent | None | 1 |
| 19 | 57 | M | None | ICA terminus | 9.9 | 3 | 1.5 | Microcatheter-first | SL-10 + Transend ES + EZ stent | 2b | Patent | None | 1 |
| 20 | 60 | F | Hypertension + Diabetes | M1 segment | 12.5 | 4 | 1.4 | Balloon angioplasty + stenting | Sinomed (2.5mm) + EP stent | 2b | Patent | Hyperperfusion syndrome (resolved) | 2 |
| 21 | 53 | M | None | ICA terminus | 8.4 | 3 | 1.6 | Microcatheter-first | Echelon-10 + Synchro + Atlas stent | 2b | Patent | None | 1 |
| 22 | 59 | F | None | M1 segment | 11.2 | 3 | 1.3 | Balloon angioplasty + stenting | Gateway (2.0mm) + EZ stent | 2b | Patent | None | 2 |
| 23 | 46 | M | None | ICA terminus | 7.6 | 2 | 1.4 | Microcatheter-first | SL-10 + Transend ES + EP stent | 2b | Patent | None | 1 |
| 24 | 65 | F | Hypertension | M1 segment | 13.6 | 4 | 1.5 | Balloon angioplasty + stenting | Sinomed (2.5mm) + Atlas stent | 2b | Patent (residual localized stenosis) | Vessel dissection (healed) | 2 |

***Notes:***

Occlusion Location: ICA = internal carotid artery; M1 = proximal middle cerebral artery.

Suzuki Collateral Grade: Assessed per the Suzuki grading system (I–VI) based on preoperative DSA.

Recanalization Strategy: "Microcatheter-first" = microcatheter advanced through occlusion followed by stenting; "Balloon angioplasty + stenting" = pre-dilation with compliant balloon prior to stent deployment.

Device Selection: Specifies microcatheter, microwire, balloon, and stent models used intraoperatively.

mTICI Grade: Modified Thrombolysis in Cerebral Infarction grade (2b = partial reperfusion, ≥50% of infarct territory; 3 = complete reperfusion).

3-Month Vascular Patency: Confirmed by DSA; "Patent" = no restenosis or re-occlusion; "Residual localized stenosis" = <50% luminal narrowing (clinically insignificant).

Complications: Documented adverse events within 3 months; hyperperfusion syndrome and vessel dissection were managed symptomatically with full recovery.

3-Month mRS Score: Modified Rankin Scale score (0 = no symptoms; 1 = minor symptoms without disability; 2 = mild disability but independent).

Pearls identified: Focal occlusion <15mm, Suzuki grade ≤4, and M2 diameter ≥1.0mm were consistent features of successful EVT; microcatheter-first approach was effective for soft occlusions, while undersized balloon angioplasty minimized vessel trauma for hard occlusions.Pitfalls identified: Patient 24 developed vessel dissection despite meeting DSA selection criteria, highlighting the need for combined HRMRI assessment of vessel wall fragility.
